# Roles of 4′-*O*-Methylalpinum Isoflavone on Activation of Microglia Induced by Oxysterols

**DOI:** 10.3390/ijms252312743

**Published:** 2024-11-27

**Authors:** Yonghae Son, Miran Kim, Dongho Lee, Ryuk Jun Kwon, Koanhoi Kim

**Affiliations:** 1 Department of Pharmacology, School of Medicine, Pusan National University, Yangsan 50612, Gyeongnam, Republic of Korea; squall0211@hanmail.net (Y.S.); alfks3051@naver.com (M.K.); 2 Department of Biosystems and Biotechnology, College of Life Sciences and Biotechnology, Korea University, Seoul 02841, Republic of Korea; dongholee@korea.ac.kr; 3 Family Medicine Clinic and Research Institute for Convergence of Biomedical Science and Technology, Pusan National University Yangsan Hospital, Yangsan 50612, Gyeongnam, Republic of Korea; 4 Department of Family Medicine, School of Medicine, Pusan National University Yangsan 50612, Gyeongnam, Republic of Korea

**Keywords:** 4′-*O*-Methylalpinum isoflavone, IL-6, inflammation, microglia, oxysterol

## Abstract

Microglia play a crucial role as immune cells responsible for the brain’s defense mechanisms. Similar to the actions of macrophages in the body, microglial cells elicit an inflammatory immune response in the brain. Recent papers highlight activated microglial cells as pivotal contributors to inflammatory responses in the brain, leading to damage to nerve tissue and the onset of Alzheimer’s disease (AD). In the brains of AD patients, elevated levels of inflammatory cytokines such as interleukin-6 (IL-6) and oxidized cholesterol metabolites (oxysterols) are observed. These factors are closely associated with inflammatory diseases in the brain. 4′-*O*-Methylalpinum isoflavone (mAI), derived from *Cudrania tricuspidata* fruit, possesses antioxidant, neuroprotective, and anti-inflammatory properties. Consequently, this study examined the effect of mAI on the expression of IL-6, a major inflammatory cytokine. The HMC3 microglial cell line was treated with oxysterols to assess the effectiveness of mAI in mitigating this inflammatory response. The results indicated that mAI inhibited the gene expression and protein secretion of IL-6 induced by 25-hydroxycholesterol (25OHChol) and 27-hydroxycholesterol (27OHChol). Furthermore, the expression of MHC class II, a marker for microglial activation, was reduced to baseline levels. These findings suggest that mAI may serve as a viable option for suppressing and treating brain inflammatory diseases induced by cholesterol oxidation products. This is achieved by curtailing the expression of the inflammatory cytokine resulting from the activation of microglial cells by immuno-oxysterol.

## 1. Introduction

Microglia are parenchymal tissue macrophages with branching processes (ramified or tree-shaped) that form about 10% of central nervous system (CNS) cells [1]. They respond to infection and injury through migration and morphological changes [2]. Activated microglia secrete inflammatory cytokines, including interleukin (IL)-1β, and increase levels of MHC class II (MHC II) molecules [3]. In the CNS, macrophages are found in the meninges, choroid plexus, and perivascular space, while microglia are derived from macrophages produced by primitive hematopoiesis in the yolk sac [4]. Gene expression and morphological changes associated with microglia activation have been widely studied. Microglia are macrophages residing in the brain and are developmentally distinct from other tissue-resident macrophage groups, remaining without a significant contribution from peripheral myeloid cells under normal conditions [5,6,7,8]. Microglial activation and neuroinflammation are prominent features of Alzheimer’s disease (AD). The activation of microglia, coupled with the release of inflammatory mediators and alterations in oxysterol profiles, could potentially amplify neuroinflammation in AD. Recently, we suggested that some side-chain oxidized cholesterols are risk factors in triggering neuroinflammatory immune responses via the activation of microglia [9].

Cholesterol is prone to oxidation, leading to the formation of oxysterols, with 25-hydroxycholesterol (25OHChol) and 27-hydroxycholesterol (27OHChol) being notable examples. These oxysterols are classified as immuno-oxysterols or immunosterols due to their ability to activate immune cells and promote the production of inflammatory mediators [10,11,12]. Several studies have demonstrated that oxysterols trigger inflammatory immune responses in both the brain and body [9,10,13,14,15,16]. Given the role of oxysterol-induced inflammation in various diseases, managing this inflammation is a significant focus in contemporary healthcare, with the discovery of anti-inflammatory agents being of paramount importance.

In this context, *Cudrania tricuspidata*, a deciduous broad-leaved thorn tree native to East Asia, including Japan, the Republic of Korea, and China, has garnered attention for its therapeutic potential. Traditionally, its root bark and cortex have been used to treat inflammation and neuritis [17]. Notably, cudraflavanone D, derived from this plant, has been shown to inhibit the production of prostaglandin E2 (PGE2), a COX-2 product, as well as the mRNA expression of key inflammatory cytokines such as IL-12, IL-1β, IL-6, and TNF-α [17]. Additionally, phenolic compounds found in various plant parts, including flowers, leaves, and bark, exhibit strong antioxidant effects with favorable safety profiles [18]. To analyze the active ingredients in more detail among these compounds, chromatographic separation was performed, and two isoflavonoids, alpinum isoflavone and 4′-*O*-methylalpinum isoflavone (mAI), were discovered through chromatographic separation of the active fraction [19,20]. However, the pharmacological activity of these isoflavone metabolites remains relatively underexplored [20].

This study aims to elucidate the effects of oxysterols, specifically 25OHChol and 27OHChol, on microglial activation and to explore whether mAI can suppress oxysterol-induced inflammatory responses. Our findings reveal a pharmacological action of mAI, characterized by its inhibition of oxysterol-induced microglial activation, as evidenced by the downregulation of pro-inflammatory cytokines. These results, along with the demonstrated anti-inflammatory properties of *C. tricuspidata*, suggest that natural compounds may provide promising strategies for mitigating oxysterol-induced inflammation.

## 2. Results

### 2.1. Expression of IL-6 Induced by Oxysterols in Microglia

**Figure 1:**
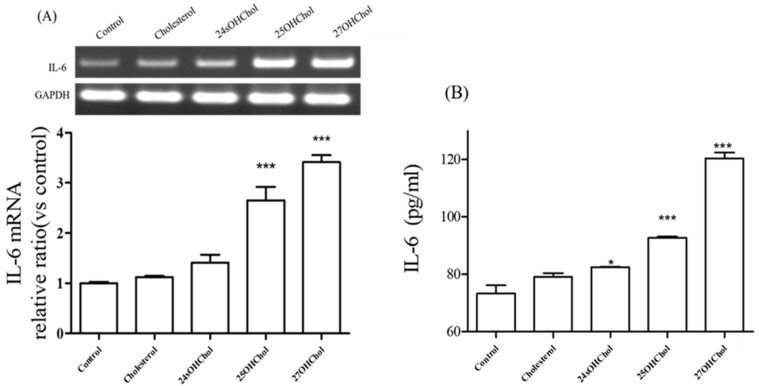
Effects of oxysterols on the expression of IL-6 in microglia. HMC3 microglial cells (1 × 10^6^ cells/well) were cultured with 1 μg/mL of cholesterol and the oxysterols in a 60 mm culture dish for 48 h. (**A**) The transcript of IL-6 was analyzed with RT-PCR and real-time PCR. The y-axis values represent fold increases in IL-6 mRNA levels normalized to GAPDH levels relative to that of the untreated microglia (control). *** *p* < 0.001 vs. control. (**B**) After separating the supernatant of the culture medium, the levels of IL-6 protein in the media were measured by ELISA. * *p* < 0.05 vs. control; *** *p* < 0.001 vs. control. Data are expressed as the mean ± SD (n = 3 replicates for each group). This experiment was independently performed three times for each condition.

To determine whether oxysterols affected the expression of IL-6 in microglial cells, the HMC3 cell line was stimulated with the indicated lipids such as cholesterol, 24sOHChol, 25OHChol, and 27OHChol. The IL-6 gene expression was assessed using RT-PCR and quantitative real-time PCR (Figure 1A). The transcript levels of IL-6 were significantly increased by the treatment with 25OHChol and 27OHChol, whereas the expression was slightly increased in the cells stimulated by 24sOHChol. And cholesterol did not influence the cytokine expression. Secretion of IL-6 was also elevated by treatment of the oxysterols, and the other lipids showed the same pattern with PCR data (Figure 1B). These findings suggest that 25OHChol and 27OHChol were able to induce IL-6 expression in microglia.

### 2.2. Effects of mAI on Oxysterol-Induced IL-6 Expression

To assess the effect of mAI on cell viability, we evaluated the toxicity of the reagent using the CCK-8 kit (Supplementary Figure S1). Treatment with mAI and oxysterols did not affect cell viability. The result indicated that mAI was not a toxic reagent.

**Figure 2:**
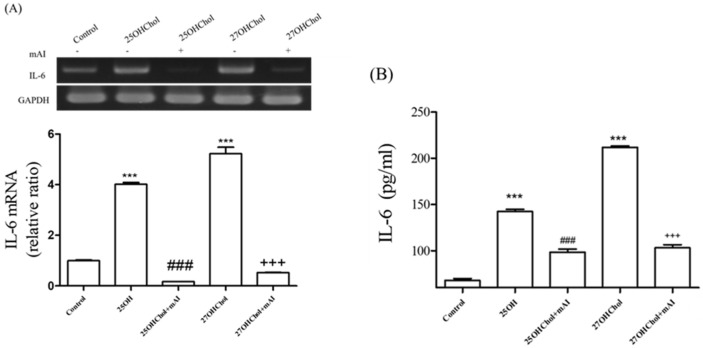
Effects of mAI on the expression of IL-6 in microglia. HMC3 cells were stimulated with 1 μg/mL of 25OHChol and 27OHChol in the absence or presence of 1 μg/mL mAI for 48 h. (**A**) Using total RNA isolated from cells, IL-6 transcripts were detected by RT-PCR and real-time PCR. The y-axis values represent increases in IL-6 mRNA levels normalized to GAPDH levels relative to that of the untreated microglia (control). *** *p* < 0.001 vs. control.; ### *p* < 0.001 vs. 25OHChol plus mAI; +++ *p* < 0.001 vs. 27OHChol plus mAI. (**B**) The levels of IL-6 protein secreted from the cells in the cultured media were measured by ELISA. The data are expressed as the mean ± SD (n = 3 replicates for each group). *** *p* < 0.001 vs. control.; ### *p* < 0.001 vs. 25OHChol plus mAI; +++ *p* < 0.001 vs. 27OHChol plus mAI. This experiment was independently performed three times for each condition.

To determine whether mAI influences the IL-6 expression induced by the oxysterol stimulation, the microglial cells were co-exposed to the oxysterols and mAI. IL-6 gene expression was upregulated by treatment with 25OHChol and 27OHChol, and the induction was completely abolished by the mAI treatment (Figure 2A). Similarly, protein secretion of IL-6, which was elevated by 25OHChol and 27OHChol, was efficiently reduced by mAI, mirroring the pattern observed at the gene expression level (Figure 2B). These findings suggest that mAI inhibits IL-6 expression induced by oxysterols, thereby attenuating microglial activation.

### 2.3. Roles of mAI on MHC II Expression of Microglial Cells Induced by Oxysterols

**Figure 3:**
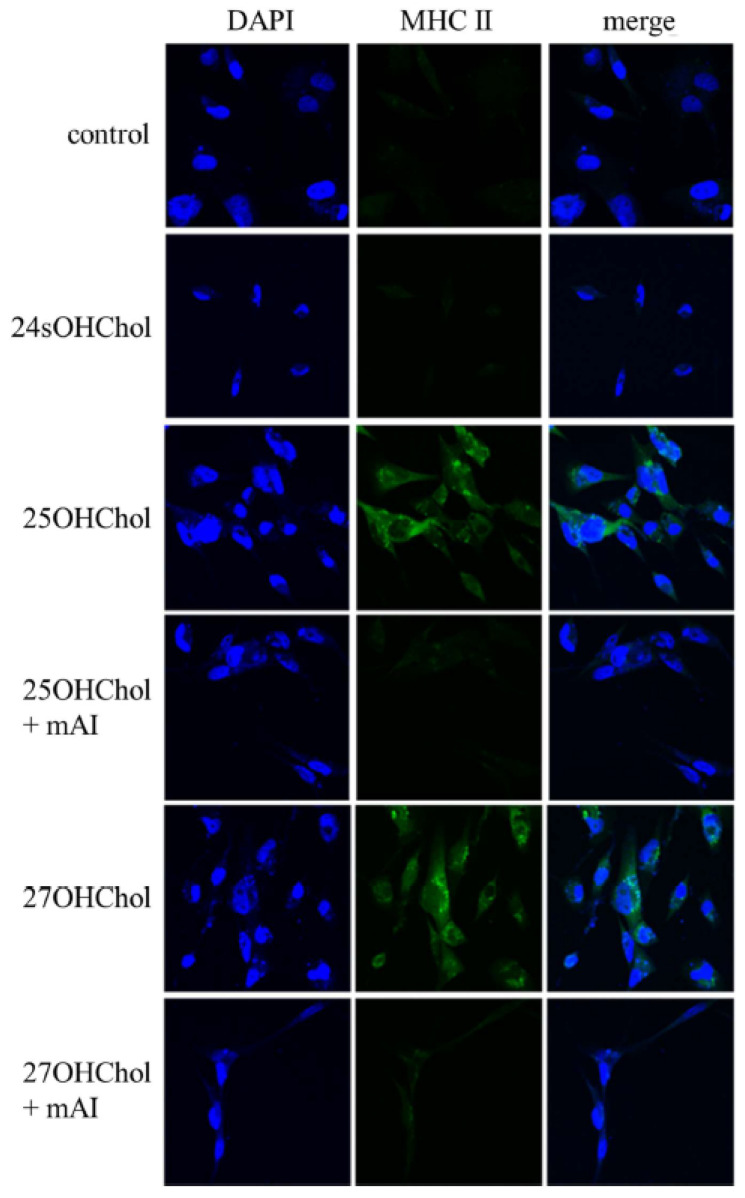
Effects of mAI on the expression of the MHC Class II on the oxysterol-stimulated microglia. The microglial cells seeded on coverslips (coated with 0.2% gelatin in PBS) were stimulated with 1 μg/mL of 24sOHChol, 25OHCho1, and 27OHChol in the absence or presence of 1 μg/mL mAI for 48 h. The cells were immuno-stained with an antibody conjugated with fluorescence for MHC II (green). The nuclei were stained with DAPI (blue) and then visualized fluorescence signals representing MHC II using a confocal microscope at 40× magnification by fixing them to slide glass. This experiment was independently performed three times for each condition.

To evaluate the impact of mAI on microglial activation induced by 25OHChol and 27OHChol, we examined the expression of MHC II, a well-established marker of microglial activation (Figure 3). A significant increase in the immunoreactivity of MHC II (green fluorescence) was observed in the microglial cells exposed to 25OHChol and 27OHChol but not in the group treated with 24sOHChol. And the treatment of mAI completely abolished these signals. These findings indicated that the reagent effectively inhibited the microglial activation triggered by the stimulation of 25OHChol and 27OHChol.

### 2.4. Effects of mAI on the Oxysterol-Induced IL-1β Expression

**Figure 4:**
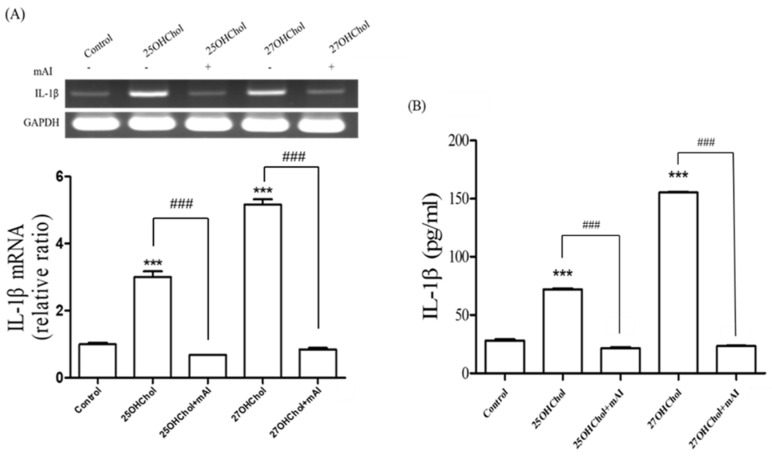
Effects of mAI on the expression of IL-1β induced by the oxysterols in microglia. The cells (1 × 10^6^ cells) were cultured in a 60 mm culture dish with 1 μg/mL of cholesterol and the oxysterols for 48 h. (**A**) Total RNA was isolated from the cells, and the transcript of IL-1β was analyzed with RT-PCR and real-time PCR. The y-axis values represent fold increases in IL-1β mRNA levels normalized to GAPDH levels relative to that of the untreated microglia (control). *** *p* < 0.001 vs. control; ### *p* < 0.001 vs. 25OHChol or 27OHChol. (**B**) After separating the supernatant of the stimulated cells, the levels of IL-1β protein in the media were measured by ELISA. *** *p* < 0.001 vs. control; ### *p* < 0.001 vs. 25OHChol or 27OHChol. Data are expressed as the mean ± SD (n = 3 replicates for each group).

We also considered the effect of mAI on IL-1β expression in the microglial cells stimulated by the oxysterols. Transcript of IL-1β was increased by the oxysterol treatment, and the induction was completely inhibited by the mAI treatment (Figure 4A). Protein secretion of IL-1β, which was up-regulated by the oxysterols, was significantly reduced by mAI in the same pattern as the gene expression level (Figure 4B). These findings indicated that mAI inhibits the IL-1β expression induced by oxysterols, thereby suppressing the microglial activation.

### 2.5. Effects of mAI on the Oxysterol-Induced Phosphorylation of Erk

**Figure 5:**
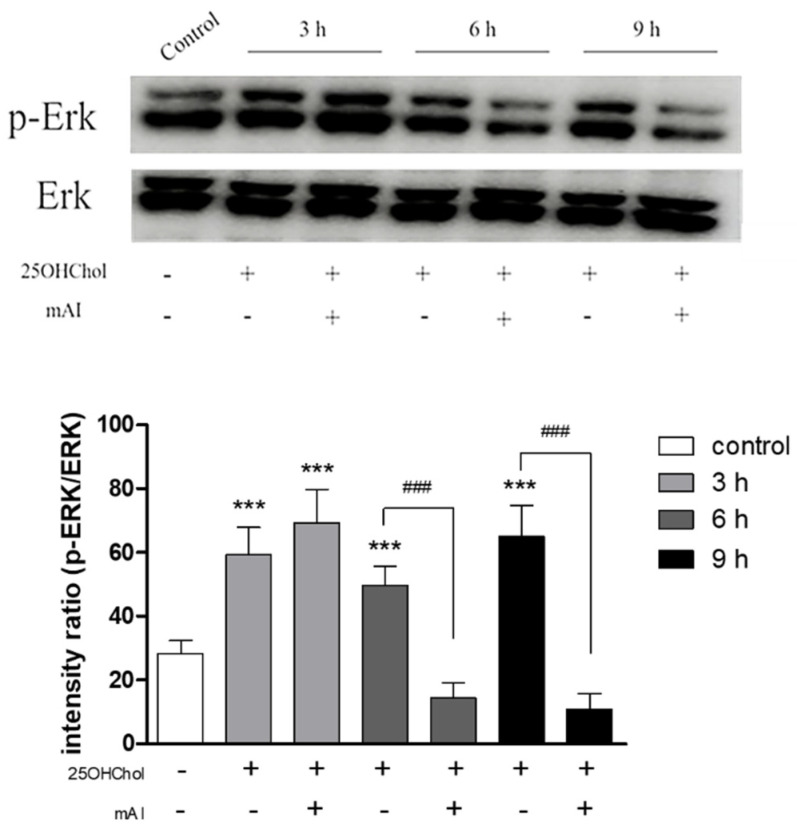
Effects of mAI on phosphorylation of Erk. The microglial cells were exposed to 1 μg/mL of 25OHChol for 3 h, 6 h, and 9 h in the absence or presence of 1 μg/mL mAI. Total proteins harvested from the cells were analyzed with immunoblotting. The figure below illustrates a graph quantifying the intensity of Western blot bands. *** *p* < 0.001 vs. control; ### *p* < 0.001 vs. the mAI treatment. Data are expressed as the mean ± SD (n = 3 replicates for each group). This experiment was independently performed three times for each condition.

We investigated the involvement of the Erk pathway in oxysterol-induced microglial activation and the effects of mAI on this pathway (Figure 5). Phosphorylation of Erk was significantly increased 3 h after the treatment of 25OHChol. However, this phosphorylation was inhibited for 6 h following the mAI treatment. These results suggested that stimulation of the oxysterol activated the Erk pathway in the microglial cells, and the activation is suppressed in the presence of mAI.

## 3. Discussion

AD is one of the leading neurodegenerative disorders associated with dementia. Pathologically, AD is characterized by aggregated β-amyloid (Aβ) plaques and neurofibrillary tangles formed by twisted τ (tau) protein filaments [21]. However, the underlying mechanisms initiating and driving AD progression remain unclear. Given the failure of numerous clinical trials targeting Aβ, current research is investigating other potential molecular pathways involved in AD. In the brain, cholesterol plays a critical role in maintaining cellular morphology, synapse formation, and neurotransmission [22]. Altered cholesterol metabolism has been identified as a significant factor in AD pathogenesis [23]. Some oxidized cholesterol metabolites perform key signaling roles and regulate cholesterol synthesis, particularly in the brain and retina [24]. Among these, 25OHChol, generated by the enzyme cholesterol-25-hydroxylase (CH25H) in the endoplasmic reticulum (ER), is particularly noteworthy [25]. While CH25H gene expression is typically low under physiological conditions, it can increase up to tenfold during inflammatory responses related to neurodegenerative diseases [16]. Macrophages, dendritic cells, and microglia are major producers of 25OHChol [16,26]. This study aimed to investigate the pathophysiological roles of oxysterols in the brain, with a focus on their effects on microglial activation. Our findings indicate that oxysterols modulate microglial activation and induce IL-6 expression, an inflammatory cytokine implicated in AD pathogenesis.

IL-6, a pivotal cytokine in immune regulation, is increasingly linked to AD. As a key mediator of inflammation, IL-6 contributes to the neuroinflammatory processes observed in AD, potentially exacerbating disease progression. Elevated IL-6 levels in the brains and cerebrospinal fluid of AD patients further suggest a strong correlation between IL-6 and AD pathology [27]. IL-6 is involved in the activation of microglia, resident immune cells of the brain, which remain chronically activated in AD, releasing various pro-inflammatory cytokines, including IL-6 itself. This positive feedback loop may promote further neuronal damage, contributing to AD progression [28]. Additionally, IL-6 influences tau phosphorylation, another hallmark of AD. Hyperphosphorylated tau forms neurofibrillary tangles that disrupt normal neuronal function. IL-6’s impact on blood-brain barrier (BBB) permeability may also facilitate the infiltration of peripheral immune cells into the brain, amplifying neuroinflammation and potentially accelerating AD progression [29]. Given IL-6’s multifaceted role in AD, it is considered a potential therapeutic target. However, clinical trials targeting IL-6 signaling in AD remain in the early stages, necessitating further research to confirm the efficacy and safety of such interventions.

In summary, IL-6 is deeply intertwined with AD pathogenesis, participating in neuroinflammation, Aβ deposition, tau phosphorylation, and BBB dysfunction. Although IL-6 presents as a promising therapeutic target, further studies are essential to fully understand its role and develop effective AD treatments. Microglial activation is triggered upon exposure to pathogen-associated molecular patterns (PAMPs) and/or endogenous damage-associated molecular patterns (DAMPs) and the removal of immunosuppressive signals [30]. Our results demonstrated that 25OHChol and 27OHChol induce the expression of MHC II, a microglial activation marker, and IL-6 production, suggesting that extracellular accumulation of 25OHChol and 27OHChol may function as DAMPs. Furthermore, we assessed whether mAI inhibits IL-1β expression, a marker of activated microglia induced by oxysterols, in microglia; our previous research showed that oxysterols induce IL-1β expression in microglia [9]. The suppression of this inflammatory cytokine supports our hypothesis that mAI inhibits oxysterol-induced hyperactivation of microglia.

Flavonoids constitute a large subgroup of phenolic metabolites in plants, with over 100 identified flavonoids exhibiting beneficial effects [31]. They are further categorized into flavones, flavanones, and isoflavones, among which certain flavonoids demonstrate potential anti-inflammatory activity. In this study, we examined the effects of mAI, the most abundant isoflavone extracted from the fruit of *C. tricuspidata*, on microglial activation. We found that mAI effectively suppresses microglial activation and the expression of IL-6 and IL-1β induced by 25OHChol and 27OHChol. Through these experiments, we validated the pharmacological mechanisms of mAI. These results indicate that mAI effectively attenuates oxysterol-induced microglial activation and the expression of IL-6 and IL-1β. Furthermore, under normal conditions, microglia exhibit low levels or no expression of MHC class II proteins on the cell surface; however, this expression becomes apparent during inflammation [32]. Despite the unclear functional significance of cellular and surface MHC II in neuroinflammatory responses in microglia [33], we performed immunofluorescence staining to confirm the effects of mAI treatment following MHC II expression. Surface molecules were detected in cells stimulated with 25OHChol and 27OHChol (green signal), and the signal was completely diminished by mAI treatment. In this study, microglia stimulated by 25OHChol and 27OHChol secreted the inflammatory cytokine IL-6, driving activation through the Erk pathway.

In conclusion, this study examined the effects of mAI, an isoflavone isolated from *C. tricuspidata*, on the response of HMC3 microglia to 27OHChol. Isoflavone inhibited the expression of inflammatory cytokines, such as IL-1β and IL-6, and the activation of microglia, resulting in anti-neuroinflammatory effects in the brain. To our knowledge, this study is the first to report that a specific type of flavonoid suppresses the activation of HMC3 microglia under conditions rich in oxidized cholesterol molecules. Although direct evidence indicating that mAI can cross the BBB remains limited, mAI, as a prenylated isoflavonoid, may interact with cell membranes and enhance BBB permeability due to its increased lipophilicity. Prenylated flavonoids, like genistein (a structurally related compound), have demonstrated BBB permeability and neuroprotective effects, suggesting that mAI may possess similar potential [19,34]. However, specific studies verifying its permeability across the BBB are still lacking, and further investigation is required to elucidate its exact capacity to cross this barrier and its potential effects on brain function.

## 4. Materials and Methods

### 4.1. Reagents

24s-Hydroxycholesterol (24sOHChol), 25OHChol, and 27OHChol were purchased from Santa Cruz Biotechnology, Inc. (Santa Cruz, CA, USA), and cholesterol and 6-diamidino-2-phenylindole dihydrochloride (DAPI) were purchased from Sigma-Aldrich (St. Louis, MO, USA). Primary antibodies for β-actin, Erk, phospho-Erk, and MHC II were purchased from Santa Cruz Biotechnology. The anti-mouse IgG-HRP and anti-rabbit IgG-HRP secondary antibodies were also purchased from Santa Cruz Biotechnology. The Alexa Fluor 488-conjugated secondary antibodies for immunofluorescence of MHC II analysis were purchased from Invitrogen (Eugene, OR, USA). mAI, a natural product, was kindly provided by Professor Dongho Lee at the Department of Biosystems and Biotechnology, Korea University, Seoul, Republic of Korea. The reagent was dissolved in dimethyl sulfoxide (DMSO; Sigma-Aldrich) and then diluted in Dulbecco’s Modified Eagle’s Medium (DMEM) for experiments.

### 4.2. Cell Culture and Treatment

HMC3 cells were obtained from the American Type Culture Collection (ATCC, Manassas, VA, USA) and cultured in DMEM supplemented with penicillin (100 IU/mL), streptomycin (10 μg/mL), and 10% fetal bovine serum (FBS) (Thermo Fisher Scientific, Waltham, MA, USA). The cells were maintained in a humidified incubator at 37 °C with 95% air and 5% CO_2_. Microglial cells at passage 7 were used for the experiments. Following an overnight incubation in DMEM containing 1% FBS, the medium was replaced, and the cells were stimulated with cholesterol or oxysterols for 48 h, with concurrent exposure to mAI.

### 4.3. Reverse Transcription (RT) and Real-Time Polymerase Chain Reaction (PCR)

Total RNA was extracted from HMC3 microglial cells using TRIsure™ reagent (Meridian Life Science Inc., Memphis, TN, USA). The RNA was then reverse-transcribed at 42 °C for 1 h in a 10 μL reaction volume, using 100 U Moloney Murine Leukemia Virus reverse transcriptase (MMLV-RT). The reaction mixture contained 50 mM Tris-HCl (pH 8.3 at 25 °C), 55 mM KCl, 3 mM MgCl_2_, 10 mM DTT, 1 μg oligo (dT)_15_ primers, 0.125 mM of each dNTP, and 40 U RNase inhibitor.

Subsequent qPCR was performed to assess the transcripts of genes of interest, and the reactions were carried out in triplicate using a CFX Maestro Real-Time PCR System (Bio-Rad, Hercules, CA, USA). Thermal cycling conditions consisted of a three-step cycling: initial denaturation at 95 °C for 2 min, followed by 40 cycles at 95 °C for 5 s, 65 °C for 30 s, and an elongation period at 72 °C for 10 s. The relative expression of each gene was calculated as the ratio to the housekeeping gene GAPDH. The primers used were as follows: IL-6 and GAPDH (Supplementary Table S1). For RT-PCR analysis, GAPDH transcripts were amplified as an internal control. The cDNA was denatured at 95 °C for 10 min, followed by 25 cycles at 95 °C for 30 s, 55 °C for 30 s, and an elongation period at 72 °C for 30 s. Primer sequences are provided in Supplementary Table S2. PCR products were size-separated by electrophoresis on 2% agarose gels for 30 min and visualized by ethidium bromide (EtBr) staining. Images were captured using the ATTO Gel Documentation System (WES-5300 CMOS, Tokyo, Japan).

### 4.4. Enzyme-Linked Immunosorbent Assay (ELISA)

The concentration of IL-6 secreted into the culture media was measured using a commercially available enzyme-linked immunosorbent assay (ELISA) kit (Human IL-6 Quantikine ELISA Kit, R&D Systems, Minneapolis, MN, USA). Following the manufacturer’s protocol, standards and samples were added in triplicate to individual wells and incubated for 2 h at room temperature. After incubation, the wells were washed three times with 1× washing buffer, followed by the addition of the conjugate solution, which was incubated for 1 to 2 h at room temperature. The wells were then washed again, and a substrate solution was added to initiate color development. After 20 min of incubation in the dark, the reaction was stopped with 2 N sulfuric acid. Absorbance was subsequently measured at 450 nm.

### 4.5. Western Blot Analysis

The microglial cells were cultured in a 60 mm dish at a density of 1 × 10^6^/mL and treated with the indicated oxysterols in the absence or presence of mAI (1 μg/mL) by incubating for 48 h. The cells were washed with 1× PBS and lysed using Pro-Prep^TM^ lysis buffer (Intron Biotechnology, Sungnam, Republic of Korea). The lysed cells were quantified using the BSA method and subjected to sodium dodecyl sulfate-polyacrylamide gel electrophoresis (SDS-PAGE) on a 12% gel. The gels were then transferred to a nitrocellulose (NC) membrane. The membranes were blocked with 5% skim milk in TBS-T buffer (0.1% Tween 20) at room temperature for 1 h. After blocking, the membranes were probed with specific primary antibodies diluted in the blocking solution (1:1000) at 4 °C overnight. The membranes were washed three times with TBS-T buffer for 20 min each and then incubated for 1 h with HRP-conjugated secondary antibodies diluted in the blocking solution (1:2000) at room temperature. After washing three times with TBS-T buffer for 15 min each, the bands were detected using a chemiluminescence reagent (Immobilon^®^ Forte Western HRP Substrate, EMD Millipore Corporation, Burlington, VM, USA). Each band was visualized using an Amersham Imager 680 (GE Healthcare, Madison, WI, USA).

### 4.6. Immunofluorescence of MHC II

HMC3 cells were cultured on gelatin-coated coverslips placed in 6-well plates (0.2% gelatin in PBS) and treated with cholesterol or oxysterol for 48 h. The cells were then fixed with 1% paraformaldehyde (PFA) for 20 min, followed by incubation in a blocking solution of 4% BSA in PBS for 1 h. Subsequently, the cells were incubated at room temperature for 4 h with a primary antibody against MHC II, diluted 1:100 in the blocking solution. After two washes with PBS, the coverslips were incubated in the dark at room temperature for 1 h with fluorescence-conjugated secondary antibodies, diluted 1:200 in 4% BSA in PBS. Finally, after another PBS wash, the fluorescence-stained cells were mounted and visualized using a confocal microscope.

### 4.7. Cell Viability

HMC3 microglial cells were seeded into 96-well culture plates at a density of 5 × 10^3^ cells per well. The viability of the microglial cells was measured after exposure to 1 μg/mL of mAI for 48 h, both in the presence and absence of 25OHChol and 27OHChol. The cell viability was determined using the Cell Counting Kit-8 (CCK-8, Dojindo, Kumamoto, Japan) reagent, which was added to each well and incubated for 2–4 h. Absorbance values at a wavelength of 450 nm were recorded using a microplate reader.

### 4.8. Statistical Analysis

All experiments were performed in triplicate or more. Unless otherwise specified, data are presented as the mean ± SD. Differences between the three groups were analyzed using one-way ANOVA, followed by Dunnett’s multiple comparison test, using GraphPad PRISM (version 5.0; GraphPad Software Inc., San Diego, CA, USA). Statistical significance was set at *p* < 0.05.

## Data Availability

All data generated or analyzed during this study are included in this published article.

## Supplementary Materials

The following supporting information can be downloaded at https://www.mdpi.com/article/10.3390/ijms252312743/s1.
