# Supplementary material for: Roles of 4′-O-Methylalpinum Isoflavone on Activation of Microglia Induced by Oxysterols
Source: Int J Mol Sci. 2024 Nov 27;25(23):12743. doi: 10.3390/ijms252312743 (PMC11641445; doi:10.3390/ijms252312743)
Supplement: Supplementary file 1 [file ijms-25-12743-s001.zip › ijms-3336400-supplementary.pdf]

Table S1. Primer sequences for real-time PCR

Primers	Sequences
Human GAPDH	Forward 5’ –GAAGGTGAAGGTCGGAGT–3’
	Reverse 5’ –GAAGATGGTGATGGGATTTC–3’
Human IL-6	Forward 5’ –CCTTCCAAAGATGGCTGAAA-3’
	Reverse 5’-TGGCTTGTTCCTCACTACT-3’

Table S2. Primer sequences for RT-PCR

Primers	Sequences
Human GAPDH	Forward 5’ –GAGTCAACGGATTTGGTCCT–3’
	Reverse 5’ –TGTGGTCATGAGTCCTTCCA–3’’
Human IL-6	Forward 5’ –CCTTCCAAAGATGGCTGAAA–3’
	Reverse 5’ –TGGCTTGTTCCTCACTACT–3’

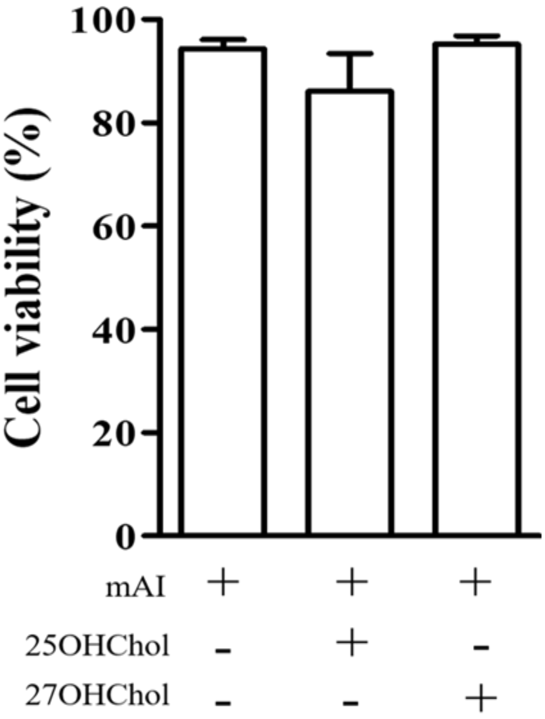

Supplementary S1. Effects of oxysterols and mAI on the viability of HMC3 microglial cells

HMC3 microglial cells were seeded in 96-well culture plates at a density of 5×10^3 cells per well in 1% FBS/DMEM medium. Subsequently, the cells were treated with 1 μg/ml of mAI or oxysterols for 48 h, after which cell viability was assessed. Data are presented as the mean ± SD (*n* = 3 replicates per group).
